# Effect of Sweet Orange Fruit Waste Diets and Acidifier on Haematology and Serum Chemistry of Weanling Rabbits

**DOI:** 10.1155/2014/421382

**Published:** 2014-04-06

**Authors:** Oluremi Martha Daudu, Rahamatu Usman Sani, Iyetunde Ifeyori Adedibu, Lawrence Anebi Ademu, Gideon Shaibu Bawa, Taiye Sunday Olugbemi

**Affiliations:** ^1^Department of Animal Science, Faculty of Agriculture, Ahmadu Bello University, Zaria 810107, Nigeria; ^2^Department of Animal Production and Health, Federal University Wukari, 641111, Nigeria

## Abstract

A total of thirty-five mixed breed (35) rabbits of average weight of 700 g aged 5-6 weeks were allocated to seven treatments in a completely randomised design to investigate the effect of sweet orange fruit waste (SOFW) and acidomix acidifier on haematology and serum chemistry. The diets were 0% SOFW, 10% SOFW with 0.5% acidomix, 10% SOFW with 0.7 acidomix, 15% SOFW with 0.5% acidifier, 15% SOFW with 0.7% acidifier, 20% SOFW with 0.5% acidifier, and 20% SOFW with 0.7% acidifier. Blood samples were analyzed for haemoglobin (hb) concentration, white blood cells (WBC), red blood cells (RBC), differential WBC count (lymphocyte, basophil, eosinophil, monocyte, and neutrophil), alanine amino transferase (ALT), alkaline phosphatase (ALP), aspartate amino transferase (AST), total protein, albumin, and globulin. There was no interaction between SOFW and acidifier for the haematological and most of the serum chemistry parameters but significant difference was observed in ALT; however the values were within the normal range. SOFW had no significant effect on all haematological and serum chemistry parameters. Acidomix had significant effect (*P* < 0.05) on haemoglobin concentration; rabbits fed 0.5% acidomix diets had higher values which were within the normal range. It is therefore concluded that SOFW with acidifier up to 20% had no detrimental effect on serum chemistry and haematology.

## 1. Introduction 

Rabbit production has a considerable potential in the developing countries for the supply of the much needed animal protein due to low capital investment and space requirement, short generation interval, rapid growth rate, high proliferation, and use of agricultural by-products Cheeke [1]. Feed is the single largest expense in livestock production which constitutes about 70% of the total cost of rabbit production Oyawoye and Nelson [2]. Maize grain is the major source of energy in rabbit feeds in Nigeria, usually accounting for over 40% of the diet [3, 4]. Rabbit production for fast meat yield is affected by inadequate and high cost of feed ingredients and brought about mainly by the stiff competition between man and monogastric animals for grain and oil seeds Agunbiade et al. [5].

A lot of research work has been conducted in Nigeria in an effort to substitute maize with cheaper and readily available ingredients in order to reduce cost and overdependence on this feedstuff for rabbit feeding. Many of these alternative feed stuffs are by-products and edible waste products from food processing, food preparation and food services industries, and bio fuel industries. Nigeria produces 3% of fresh citrus in the world and Africa produces 3,741,000 ton of different varieties of citrus fruits of which Nigeria contributes 3,240,000 ton FAO [6]. A lot of the orange harvested is wasted due to few and small capacity of the processing industries to convert the fruit to juice, concentrate, and canned fruit Hon et al. [7]. It constitutes an environmental challenge since it is not being put into productive use. The excess can be utilized for feeding of livestock such as rabbits that can handle high fibre diets.

Organic acids have been used for decades in feed preservation, protection of feed ingredients from microbial and fungal deterioration, or improving the shelf life of fermented feed Canibe et al. [8]. It also has the capacity to reduce pH and the feed's buffering capacity and its antimicrobial effect helps prevent the growth of bacteria and kills microorganisms. The acidifier used in the study was Acidomix AFG. It is a microgranulated feed acidifier based on formic acid (E-236), propionic acid (E-280), ammonium formate (E-295), and ammonium propionate (E-284) adsorbed to a silica carrier (E-551a).

Dried citrus pulp is susceptible to moulds due to its hygroscopic capacity particularly in humid tropical climates. These moulds produce secondary metabolites such as aflatoxins and citrinin (the latter is known to cause hemorrhagic syndrome) [9]. Contamination with pesticide residues can also occur and depends on the compound, dose used, amount of rain, time between application and harvest, and Citrus species [9]. Blood analysis determines explicit states of stress which can be management; breed of an animal, environment, and physiology of the animal. Haematological indices are generally used to determine the health condition of animals generally Kamal et al. [10]. This study aimed at determining the effect of feeding varying levels of sweet orange fruit waste meal and acidomix© diets on the haematology and serum chemistry of rabbits.

## 2. Materials and Methods

### 2.1. Experimental Site

The experiment was conducted at the research farm unit of the Department of Animal Science, Ahmadu Bello University, Samaru, Zaria, Kaduna State. Zaria is within the Northern guinea savannah zone of Nigeria, latitude 11°12′N and longitude 7°33′E, at an altitude of 610 m above sea level.

### 2.2. Source and Processing of the Sweet Orange Fruit Waste (SOFW)

The sweet orange fruit waste used in the experiment consisted of discarded sweet oranges gathered from traders at the Railway station market in Kaduna State. The unpeeled oranges were washed, split-open, sun-dried, stored in polythene bags until they were milled, and incorporated into the experimental diets. Most of the seeds were removed during the drying process to reduce the limonene content of the fruit.

### 2.3. Experimental Diets

The diets consisted of the sweet orange fruit waste at graded levels of 0%, 10%, 15%, and 20% and two levels of Acidomix AFG (0.5% and 0.7%) as shown in Table 1. The diets were formulated to meet the nutritional requirements for weaner rabbits.

### 2.4. Management of Experimental Animals and Data Collection

A total of 35 mixed breed weanling rabbits aged 5-6 weeks with average weight of 700 g were used for the study, each treatment consisted of five rabbits. The experimental design was 2 × 2 factorial arrangement, in a completely randomized design. There were seven treatments with graded levels of the sweet orange fruit waste treated with Acidomix AFG at two levels. The animals were kept individually in cages equipped with feeding and drinking troughs; feed and water were administered ad lib. The cages had wire screen bottoms, which allowed faeces and urine to pass into a collection grid; hence the rabbits had little contact with their voided faeces and urine. The rabbits were subjected to a two-week adjustment period before the trial commenced. The experiment lasted for 56 days.

### 2.5. Blood Analyses

The rabbits were fasted for 14 hours prior to blood analysis. The rabbits fasted for 14 hours prior to blood collection. During blood collection, 1 mL of blood was collected via the ear vein of the rabbits into sample bottles containing Ethylene Diamine Tetra acetic Acid (EDTA) to prevent clotting of blood for haematological analysis and 2 mL was collected in a plain bottle for serum chemistry analysis. The total red blood cell (RBC) and white blood cell (WBC) count were determined using improved haemocytometer method as described by Lamb [11]. Differential WBC count was determined by preparing blood smear stained with Wrights stain as described by Ross et al. [12]. Haemoglobin concentration was estimated using cyanomethaemoglobin method as described by Jain [13].

Blood samples collected in the plain test tubes were centrifuged at 3,000 revolutions per minute (rpm) for 10 minutes and the serum was collected and stored at −20°C until analyzed for alanine aminotransferase (ALT) and aspartate aminotransferase (AST) using Reitman and Frankel Method, alkaline phosphatase (ALP) was determined using Beckman Synchron method. Total Proteins (TP) was determined using Biuret method as described by Reinhold [14], albumin values were obtained by bromocresol green method as described by Doumas and Biggs [15], and globulin values were determined according to the method of Coles [16].

### 2.6. Data Analyses

Data were subjected to analysis of variance, using the General Linear Model (GLM) procedure of Statistical Analysis System [17]. Difference between treatment means was separated using Duncan multiple range test.

## 3. Results and Discussion

Table 2 shows the proximate composition of sweet orange fruit waste (SOFW). The dry matter was 93.46%, crude protein was 6.44%, crude fibre was 4.89%, and ash was 4.01%. The minerals were sodium 0.68%, potassium 0.92%, total phosphorus 0.11% calcium 0.62%, and magnesium 0.15%. Metabolizable energy was 4030 kcal/kg.

Table 3 shows the effect of SOFW on haematology and serum chemistry of weaner rabbits. There was no significant difference (*P* > 0.05) observed in WBC values; the values fell within the range of 5–13 × 10^9^as reported by Chilson [18]. There was no significant difference (*P* > 0.05) observed in RBC across the treatments' the values obtained fell within the normal range of 3.8–7.9 × 10^6^/mm^3^ as reported by Chilson [18]. There was no significant difference (*P* > 0.05) in lymphocyte count (39.7–41%), the values were within the normal range (40–80%) as reported by RAR [19]. There was no significant difference (*P* > 0.05) for eosinophil (7.9–8.5%), but the values were higher than the normal range of 0–4% as reported by Research Animal Resources [19]. The increase in eosinophils is likely due to allergy and respiratory or gastrointestinal disease Ganong [20]. There was no significant difference (*P* > 0.05) observed in monocyte (8.37–8.92%); the values were higher than normal range of 1–4% [16]. The primary function of monocytes is their role as critical immune effector cells that respond to signals from both innate and antigen-specific immune cells. They also contribute to wound healing and immune regulation. Monocytes carry out phagocytosis to protect the organism from harmful pathogens and to remove dead, dying, or damaged cells from the blood. The observed increase is likely due to the presence of foreign organisms such as coccidia which needed to be eliminated from the body. There was no significant (*P* > 0.05) difference in basophil across the treatments; the values (4.17–4.56%) were within the normal range of 1–7% reported by [19]. There was no significant difference (*P* > 0.05) in neutrophil count across the treatments; the values fell within the normal range of 34–70% (http://www.medirabbit.com/). There was no significant difference (*P* > 0.05) for globulin across the treatments as reported by [18]. Slightly higher values were observed for rabbits fed 10% SOFW diet. The normal range is 25–40 g/L, reported by Chilson [18]. Globulins are carrier proteins for steroid and thyroid hormones and play a vital role in natural and acquired immunity to infection [20]. The increase in globulins observed could be attributed to the presence of an infection or due to individual differences in the rabbits fed this diet since no increase was observed for rabbits fed higher levels of SOFW diet. There was no significant difference (*P* > 0.05) for AST values; the values fell within the normal range [18]. There was no significant difference (*P* > 0.05) for ALP; the values fell within the normal range of 10–96 IU/L [18]. There was no significant difference (*P* > 0.05) observed across the treatment for total protein; the values fell within the normal range of 50–75 g/L. There was no significant difference (*P* > 0.05) observed in albumin; the values also fell within the normal range of 25–40 g/L [18].

Table 4 shows the effect of acidomix AFG on haematology and serum chemistry. There was no significant difference (*P* > 0.05) in the WBC count. The values (4.8–5.5 × 10^9^) were similar to the normal range for WBC (5–13 × 10^9^) as reported by Chilson [18]. There was no significant difference (*P* > 0.05) in RBC count; the values were within the range of 3.8–7.9 × 10^6^/mm^3^ as reported by Chilson [18]. There was no significant difference (*P* > 0.05) in lymphocyte (40.3–40.6%) but values obtained were a bit lower than the normal range of 43–80% as reported by Chilson [18]. There was no significant difference (*P* > 0.05) in the eosinophil value (8.23%) but it was higher than the normal range of 0–2% as reported by Chilson [18]; this is an indication that the animals were possibly fighting an infection. There was no significant difference (*P* > 0.05) observed in Basophil (4.26–4.53%) but the values were higher than the normal range of 0–0.84% as reported by Chilson [18]. A combination of eosinophilia and basophilia is observed in allergic based inflammation, parasitic infestation, skin and respiratory inflammation that is allergic in nature. Postmortem reports showed presence of coccidian; this can account for increase in eosinophil and basophil [21]. There was no significant difference (*P* > 0.05) in neutrophil and it fell within the normal range of 34–70% as reported by Chilson [18]. A significant difference (*P* > 0.05) was observed across the treatments for haemoglobin (12.54–13.28 g/dL) but it fell within the normal range of 9.4–17.4 g/dL as reported by Chilson [18]. There was a significant difference (*P* > 0.05) in the AST value. Rabbits that fed 0.7% acidifier diets had higher AST level (18.68 u/L) but the values fell within the normal range of 10–98 u/L as reported by Chilson [18]. There was no significant difference (*P* > 0.05) in the value of ALT (31.15–34.68 u/L) across the treatments, but it fell below the normal range (55–260 u/L) as reported by Chilson [18]. There was no significant difference (*P* > 0.05) across the treatments for ALP; values fell within the range (10–96 u/L) as reported by Chilson [18]. There was no significant difference (*P* > 0.05) for total protein across the treatments; it lies within the normal range 25–40 g/L as reported by Chilson [18]. There was no significant difference for globulin (35–38.79 g/L) but values obtained were higher than the normal range (15–33 g/L) as reported by Chilson [18].

**Figure 1:**
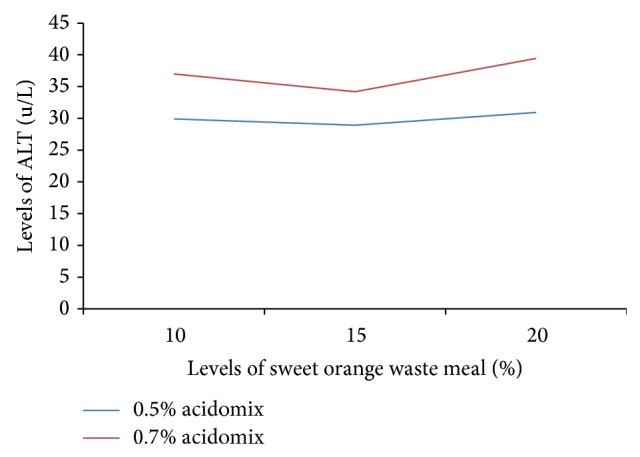
The effect of sweet orange fruit waste and acidifier on the Alanine amino transferase of weanling rabbits.

Figure 1 shows the effect of SOFW and acidifier on the haematology and serum chemistry of weaner rabbits. There was interaction between SOFW and acidifier; an increase was observed in ALT level for all levels of SOFW with increase in the level of acidifier. The greatest difference in slope was observed between 0.5% and 0.7% at 20% SOFW and is the strongest point of interaction. There was a significant difference (*P* > 0.05) across the treatments for ALT values; higher values were observed with the inclusion of 0.7% acidomix but the values obtained were within the range (http://www.medirabbit.com/). The increase in ALT levels could be attributed to Acidomix damaging the liver thus causing leakage of the enzyme out of the liver.

## 4. Conclusion

The inclusion of SOFW and acidomix in the diet of weaner rabbits did not affect haematological and serum chemistry parameters; hence it can be fed to weaner rabbits.

## Figures and Tables

**Table 1:** Composition of experimental diets fed to weaner rabbits.

Ingredients (Kg)	0% control	10% + 0.5% acidomix	10% + 0.7% acidomix	15% + 0.5% acidomix	15% + 0.7% acidomix	20% + 0.5% acidomix	20% + 0.7% acidomix
Maize	45.95	35.26	35.26	29.90	29.90	24.55	24.55
Soyabean meal	25.20	25.39	25.19	25.75	25.55	26.10	25.90
SOFW	—	10.00	10.00	15.00	15.00	20.00	20.00
Palm kernel cake	10.00	10.00	10.00	10.00	10.00	10.00	10.00
Rice offal	15.00	15.00	15.00	15.00	15.00	15.00	15.00
Bone meal	3.00	3.00	3.00	3.00	3.00	3.00	3.00
Salt	0.30	0.30	0.30	0.30	0.30	0.30	0.30
Acidomix AFG	—	0.50	0.70	0.50	0.70	0.50	0.70
Vitamin premix	0.25	0.25	0.25	0.25	0.25	0.25	0.25
Methionine	0.20	0.20	0.20	0.20	0.20	0.20	0.20
Lysine	0.10	0.10	0.10	0.10	0.10	0.10	0.10
Total	**100.00**	**100.00**	**100.00**	**100.00**	**100.00**	**100.00**	**100.00**
Calculated analyses							
Crude protein (%)	18.00	18.00	18.00	18.00	18.00	18.00	18.00
Ether extract (%)	4.52	5.86	5.86	6.53	6.53	7.21	7.21
Crude fibre (%)	10.15	10.46	10.46	10.62	10.62	10.78	10.78
Calcium (%)	0.89	0.89	0.89	0.89	0.89	0.89	0.89
ME (Kcal/Kg)	2604	2651	2651	2675	2675	2699	2699
Feed cost (₦/kg)	68.31	70.07	72.85	67.48	70.26	64.89	67.67

0.25 kg of premix will supply the following: vitamin A 1500 IU, vitamin D 300 IU, vitamin E 3.00, vitamin K 0.25 g, thiamine 0.2 mg, riboflavin 0.6 mg, pantothenic acid 1.00 mg, pyridoxine 0.4999 mg, niacin 4.00 mg, vitamin B12 0.002 mg, folic acid 0.10 mg, biotin 0.008 mg, choline chloride 0.05 g, antioxidant 0.012 g, manganese 0.0096 g, zinc 0.0060 g, copper 0.0006 g, iodine 0.006 g, iodine 0.00014 g, selenium 0.024, and cobalt 0.004 mg.

**Table 2:** Proximate composition of sweet orange fruit waste.

Nutrient	Sweet orange fruit waste (SOFW)
Dry matter (%)	93.46
Crude protein (%)	6.44
Crude fibre (%)	4.89
Ether extract (%)	17.00
Ash (%)	4.01
Nitrogen free extract (%)	67.66
Neutral detergent fibre (%)	19.11
Acid detergent fibre (%)	17.75
Minerals	
Na (%)	0.68
K (%)	0.92
Total Phosphorus (%)	0.11
Ca (%)	0.62
Mg (%)	0.15
^1^Metabolizable energy (Kcal/kg)	40.30

^1^(37 × %CP) + (81.8 × %EE) + (35.5 × %NFE); Pauzenga (1985).

**Table 3:** The effect of sweet orange fruit waste on haematology and serum chemistry of weaner rabbits.

Parameter	Levels of sweet orange fruit waste (%)				SEM
	0	10	15	20	
WBC (×10^9^/L)	5.05	7.80	5.08	5.69	0.105
RBC (×10^12^/L)	4.09	5.91	5.14	5.27	0.045
Lymphocyte (%)	39.74	40.99	40.36	40.08	0.006
Eosinophil (%)	7.89	8.11	8.17	8.51	0.022
Monocyte (%)	8.92	8.71	8.37	8.82	0.009
Basophil (%)	4.53	4.47	4.17	4.56	0.015
Neutrophil (%)	44.23	44.27	44.65	43.48	0.007
Haemoglobin (g/dL)	12.20	13.03	13.15	13.08	0.013
AST (u/L)	15.72	16.10	20.24	18.90	0.038
ALT (u/L)	29.46	31.95	33.83	33.77	0.024
ALP (u/L)	72.36	71.54	76.16	77.19	0.029
Total protein (g/L)	72.89	74.09	69.74	70.48	0.024
Albumin (g/L)	32.99	39.65	39.39	39.89	0.019
Globulin (g/L)	30.22	41.57	33.55	40.64	0.052

AST: aspartate amino transferase, ALT: alanine amino transferase, ALP: alkaline phosphatase, RBC: red blood cells, WBC: white blood cell.

**Table 4:** The effect of acidomix AFG on haematology and serum chemistry of weaner rabbits.

Parameter	Levels of acidomix (%)		SEM
	0.5	0.7	
WBC (×10^9^/L)	5.52	4.81	0.01
RBC (×10^12^/L)	5.20	5.23	0.06
Lympocyte (%)	40.22	40.57	0.01
Eosinophil (%)	8.23	8.23	0.01
Monocyte (%)	8.44	8.81	0.01
Basophil (%)	4.53	4.26	0.02
Neutrophil (%)	44.58	43.56	0.03
Haemoglobin (g/L)	13.28^a^	12.54^b^	0.02
AST (u/L)	17.39	18.68	0.05
ALT (u/L)	31.15	34.68	0.03
ALP (u/L)	70.84	80.00	0.04
Total protein (g/L)	70.96	72.51	0.03
Albumin (g/L)	38.60	38.63	0.03
Globulin (g/L)	38.79	35.00	0.07

^ab^Means with different superscripts within a row differ significantly (*P* < 0.005), AST: aspartate amino transferase, ALT: alanine amino transferase, ALP: alkaline phosphatase, RBC: red blood cell, WBC: white blood cell.
